# Ultrasound imaging versus morphopathology in cardiovascular diseases. Coronary atherosclerotic plaque

**DOI:** 10.1186/1476-7120-2-29

**Published:** 2004-12-14

**Authors:** Giorgio Baroldi, Riccardo Bigi, Lauro Cortigiani

**Affiliations:** 1 Institute of Clinical Physiology, National Research Council, Milan and Pisa, Italy; 2 University School of Medicine and "A. De Gasperis" Foundation, Niguarda Hospital. Milan, Italy; 3 Cardiovascular Unit, "Campo di Marte" Hospital, Lucca, Italy

## Abstract

This review article is aimed at comparing the results of histopathological and clinical imaging studies to assess coronary atherosclerotic plaques in humans. In particular, the gap between the two techniques and its effect on the understanding of the pathophysiological basis of coronary artery disease is critically discussed.

## Introduction

Amongst the clinical approaches ultrasound imaging is one of the more promising technique to understand dysfunction. The need is to compare morphopathological counterpart to have a correct pathophysiological interpretation.

In four reviews, morphopathology of the main cardiovascular disorders, in relation to the status of art of clinical imaging will be presented. The aim is to recall the pathological anatomy to stimulate ultrasound experts to further sharpen their technology till "histological" perfection.

The present first review concerns the coronary atherosclerosis since the current dogma of "unifying theory" assumes that the acute coronary syndromes, namely unstable angina, myocardial infarct and sudden death, are caused by atherosclerotic plaque rupture or "explosion" with occlusive thrombus formation preceded by intramyocardial emboli. An assumption which implies to discover a clinical imaging able to show when a coronary atherosclerotic plaque becomes vulnerable i.e. prone to rupture.

The risk of the latter has been correlated with large lipid core (atheroma in our definition), thin fibrous cap (< 65 μm) covering atheroma. Therefore any imaging should have a 50 μm resolution to identify a fibrous cap prone to rupture, 100 μm or 150 μm thickness being respectively at low or minimal risk. Matrix-digesting enzymes released from inflammatory cells (monocytes, macrophages, T-cells, B-cells, neutrophils, mastcells) may contribute to plaque rupture. An attractive approach since, despite many years of preventive and therapeutical attempts, coronary heart disease (CHD) remains the main cause of death and morbidity in advanced societies and selects people at the top of their work skillness and productivity.

The first question is whether ultrasound imaging may or may not discriminate extent and morphology of plaque variables seen within the intima.

## Coronary atherosclerotic plaque

### Physiological intimal thickening

**Figure 1:**
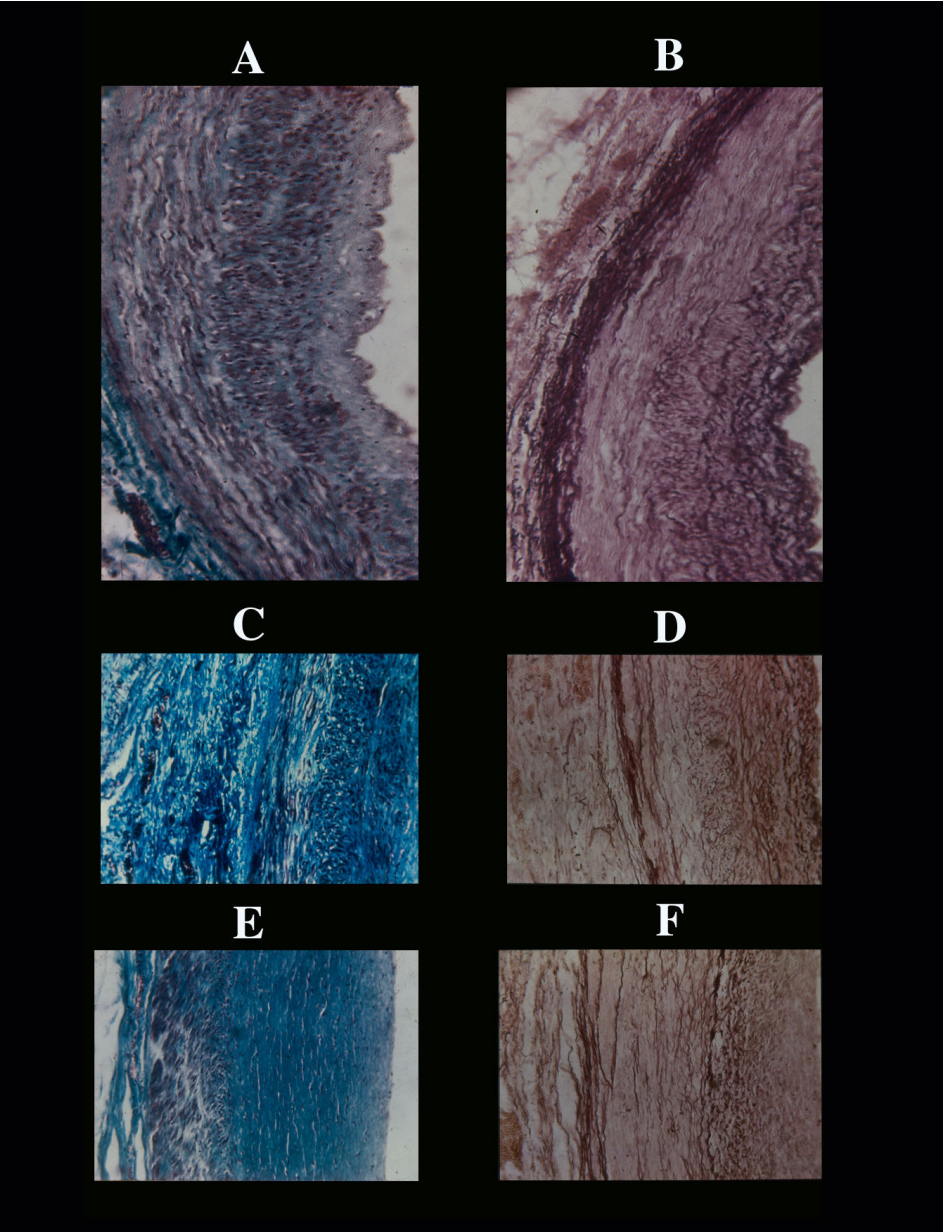
Coronary physiologic intimal thickening. This changes starts as nodular (already visible at birth at the site of vessel bifurcation) smooth muscle cells (A) and elastic fibrils (B) hyperplasia which in the second decade is diffuse to the whole intimal surface of all extramural arterial vessels. With aging there is a progressive increase of fibrous tissue which substitutes myo-elastic tissue (C, D) with final total, anelastic, fibrosis (E, F). Arteriosclerosis distinct from atherosclerosis

**Figure 2:**
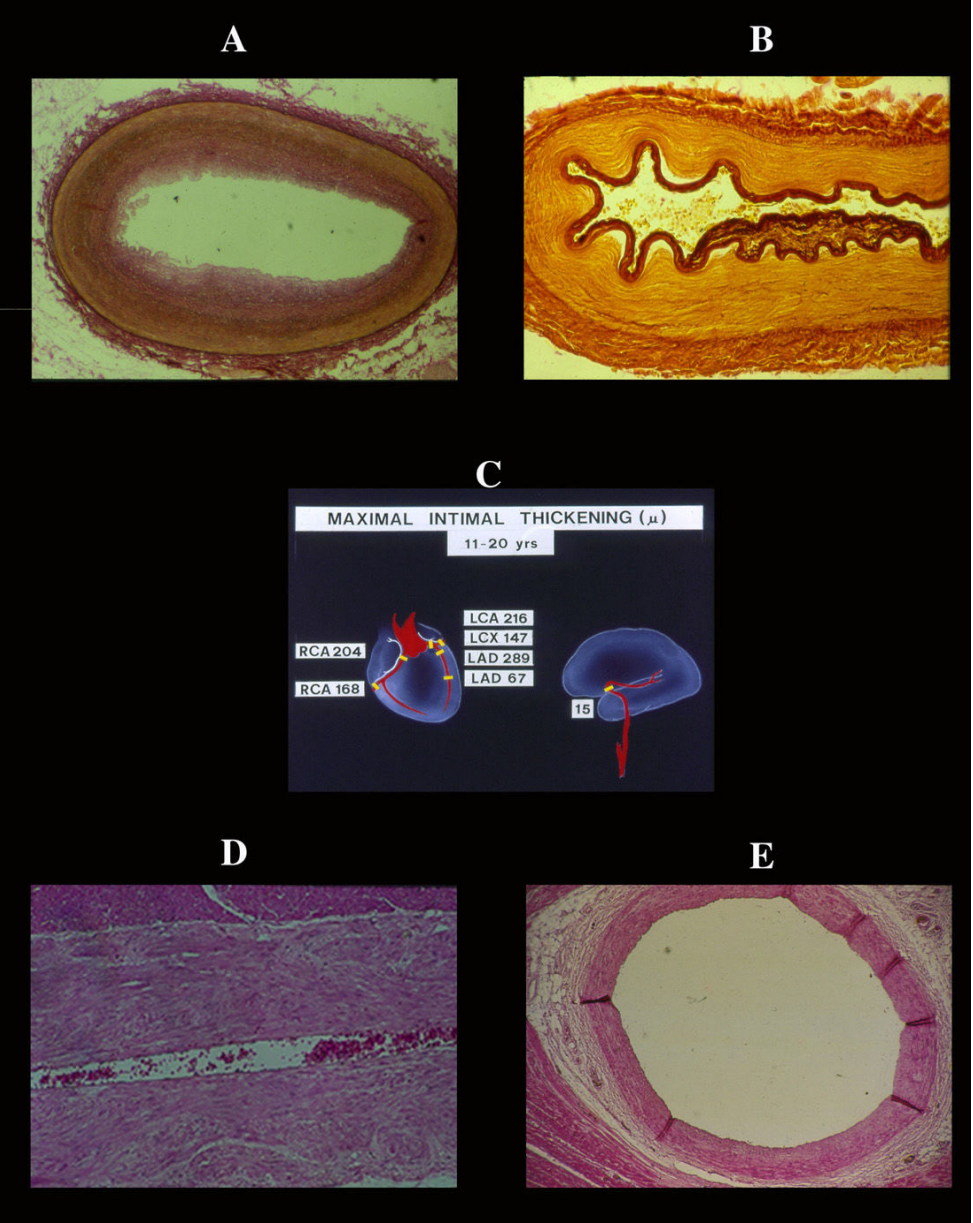
Comparison between the intimal thickening of the LAD (A) and the middle cerebral artery (B) of the same 18-year old subject. In the latter artery the intimal thickening is minimal in contrast to that of LAD which is circumferential with a thickness greater than tunica media. C), difference in maximal thickness in microns found in main coronary arteries and branches in respect of the middle cerebral artery. D), absence of intimal thickening in the LAD of dogs, despite and identical morpho-function. This suggests a possible role of the neurogenic control of coronary arteries in humans. On the other hand the absence of intimal thickening in the "mural tract" of coronary arterial vessels (E) emphasizes the role of systolic dynamic stresses on arterial wall free to expande versus those protected by encircling contracted myocardium.

Morphology of the atherosclerotic plaque has been described in textbook and articles [1-4]. In comparing many contributions, the major difficulty is to discriminate among different morphologic patterns selected in different groups of patients according to unclear definitions and without distinction between the plaque obtained by hypercholesterol diet and that found in the general population. The other need is to consider the evolution of a plaque in each single arterial system since anyone has its own peculiarity with different response to blood flow dynamics. In this sense, only the coronary arteries have a diphasic blood flow in relation to the cycle of myocardial contraction, i.e. filling of extramural vessels without intramural flow in systole because contracted myocardium compresses intramyocardial vessels. The result is an excess of systolic radial, circumferential, longitudinal and drag pressures on the wall of arteries and branches free to expand on the cardiac surface. In turn, this diphasic hemodynamic induces a structural response of the coronary intima which starts as smooth muscle cell proliferation from the tunica media, followed by elastic fiber hyperplasia ending in fibrosis of the whole intima without lumen reduction (Fig 1). First described by Wolkoff in 1929, such thickening becomes greater in the second decade in contrast to its absence in other human muscular arteries (e.g. brain arteries) or in animal with a similar coronary artery anatomy and diphasic flow (Fig. 2). The latter finding suggests that hemodynamic pressures may have the intimal hyperplastic effect in association with the neurovegetative regulation of arterial wall tone particularly active in humans in relation to heart function. Any clinical imaging of the coronary wall should consider this *physiological intimal thickening *[3-5] which in normal adult hearts measures about 200 μm, does not show any atherosclerotic variable, may become thicker in hypertrophic hearts with normal coronary artery and is greatly reduced or absent in segments of extramural coronary artery embedded ("mural artery") within the myocardium. The latter abolishes the systolic arterial wall expansion (Fig. 2).

The first conclusion is that such intimal thickening is a physiologic structural respons to hemodynamics and not the initial phase of atherosclerosis as claimed by some authors.

#### Coronary atherosclerotic intimal thickening

In contrast to the uniformely diffuse physiological intimal thickening, the atherosclerotic one is focal and protrudes within the lumen which is progressively reduced. In order to quantitatively study this progression, we sampled systematically in each heart the first tract of the main left trunk (LCA), left descending (LAD) and circumflex (LCX) branches, right coronary artery (RCA), posterior descending branch (PD) and the middle tract of LAD and marginal and posterior tracts of RCA. These selected tracts correspond to the sites where atherosclerotic changes generally occur. The coronary arterial sampling was performed in 100 fatal cases of acute myocardial infarct without other diseases and not undergone invasive techniques; 208 cases of sudden and unexpected coronary death (SUD) which occurred in apparently normal people, acting their usual life, without resuscitation attempts and autopsy findings limited to coronary atherosclerosis of any degree, myocardial necrosis or scar, with or without cardiac hypertrophy: 50 cases with chronic angina pectoris who died within 25 day after coronary by-pass surgery; and 97 normal subjects who died by accident without pathological findings at autopsy but coronary atherosclerosis. In a total of 3,640 coronary sections the following variables were quantified:

1. *Lumen reduction *calculated in percent of the normal diameter measured in normal coronary arteries and branches injected by plastic substance under pressure. Measurement often referred to the cross-sectional area within the internal elastic membrane may result in severe stenosis despite a normal lumen since the atherosclerotic plaque may enlarge the cross-section.

2. *Shape *of residual lumen: concentric if encircled by pathological intima or semilunar when an arch of the wall was normal.

3. *Length *calculated in number of segments involved by the plaque, all extramural coronary arteries being sistematically cross-sectioned at 3 mm interval.

4. Intimal and tunica media thickness measured in microns.

5. *Atherosclerotic changes *within the intima: *fibrosis *alone, *basophilia *i.e. proteoglycan accumulation, *atheroma *or lipoprotein/cholesterol material, *calcification*, *vascularization*, *hemorrhage*, *adventitial *and *intimal lymphocytic infiltration*. All these variables were expressed in percent of the total intima but vascularization calculated in number of vessels found.

Amongst 1,519 sections without lumen reduction with an intimal physiological thickness less of 300 μm we never found subendothelial or internal lipoprotein/cholesterol infiltration or deposit (fatty streaks), monocytes or macrophages or foam cells, platelet aggregates, fibrin-platelet thrombi or inflammatory elements. A similar negative finding was observed in 1,315 coronary sections with a lumen reduction less than 69% and pathological intimal thickness less than 600 μm. In all these sections we were unable to demonstrate an intimal fissuration. We must emphasize that in selecting our material cases of familial hypercholesterolemia were excluded.

In general the atherosclerotic intimal variables increased in frequency and extent in parallel with the lumen reduction and pathological intimal thickeness with the exception of proteoglycan accumulation less found in stenoses >90% and intimal thickness >2000 μm. Of 990 sections with calcification, 488 (49%) had mild stenosis, calcification being severe in 162 (33%). This finding indicates that calcification per se does not necessarily means a severe lumen reduction. The less frequent variable was intimal hemorrhage mainly seen in plaques located in a vessel tributary to an acute infarct.

**Figure 3:**
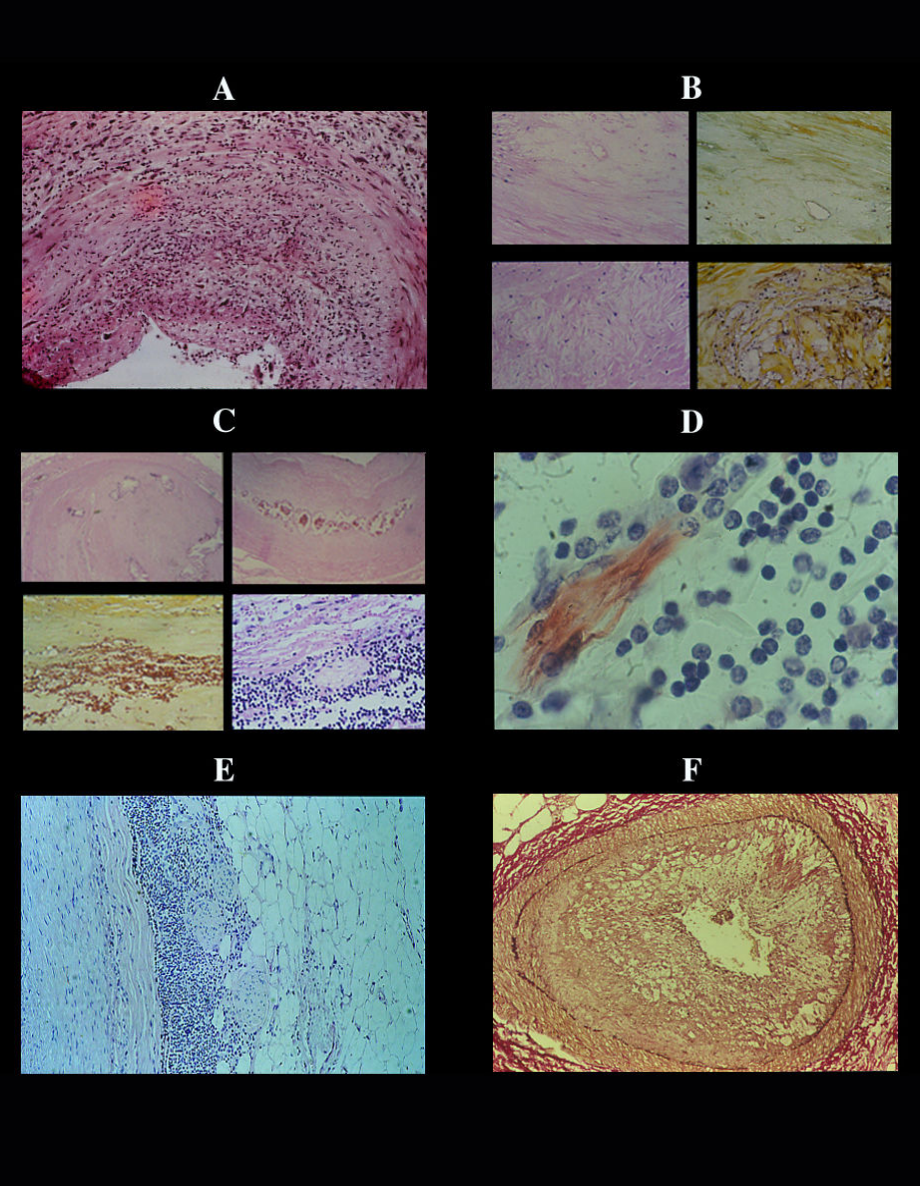
Natural history of the coronary atherosclerotic plaque in general population, including most of CHD patients. The starting point is a nodular hyperplasia of smooth muscle cells and elastic tissue with progressive fibrous replacement. No other changes as subendothelial lipo-protein-cholesterol storage, inflammatory process of any type, platelet aggregation and/or fibrin-platelet thrombi are found (A). Proteoglycan accumulation in the deep intima between tunica media and the fibrous cap is the second step (B). In this proteoglycan pool, lipo-protein/cholesterol cleft, in macrophages ("foam cells") and/or Ca^++ ^salts appear. Vascularization of the plaque and hemorrhage (C) follow. In the stage of proteoglycan accumulation, lympho-plasmacellular infiltrates occur in the adventitia and intima (C) with specific localization, around adventitial nerves closed to the tunica media (medial neuritis) (D, E). This natural history is totally different from that obtained experimentally by hypercholesterol diet in animals free of spontaneous atherosclerosis or in the small group of patients with familial hypercholesterolemia (F), in which transendothelial lipo-protein insudation is the starting point.

When different groups of CHD and normal people were matched, an excess of atheroma, hemorrhage, calcification and adventitial/intimal lymphocytic inflammation was observed in AMI group while a significant defect was present in normal subjects with the same degree of stenosis. In synthesis, two other main findings are worth of mention: 1) proteoglycan accumulation is a relatively late event which occurred in the deep layer of the intima near to the tunica media and below the fibrous cap of a plaque. Lipoprotein/cholesterol plus macrophages (foam cell) and/or calcium salts appeared only in this proteoglycan pool in agreement with their interaction with glycosaminoglycans (Fig. 3); 2) adventitial inflammation showed a peculiar tropism for the nervous structures related to the media at plaque level only (medial neuritis). An inflammatory process which involved all plaques present in each CHD patient while absent or limited to one plaque in normal controls.

This study induced the recognition of two types of coronary atherosclerotic plaque: one, which belongs to the general population, (including CHD patients) and starts as nodular intraluminal proliferation of smooth muscle cells followed by elastic tissue hyperplasia and final substitution by fibrous tissue. Subsequently, a deep proteoglycan pool forms and becomes a deposit of lipoprotein/cholesterol and/or calcium salts. The recurrence of these phenomena explains the radial, circumferential, longitudinal progression of the coronary plaque resulting in increasing lumen reduction. This type of *myohyperplastic plaque *is totally different from the *hypercholesterol plaque *obtained experimentally by hypercholesterol diet in animals free of atherosclerosis or observed in a small group of patients with familial hypercholesterolemia. In literature too often the hypercholesterol plaque is taken as a model of an atherosclerotic plaque in man [1]. In timing the sequence of the events is important to stress that the inflammatory lymphocytic-plasmacellular process (autoimmune phenomenon?) starts after the proteoglycan insudation, being a relatively late complication.

**Figure 4:**
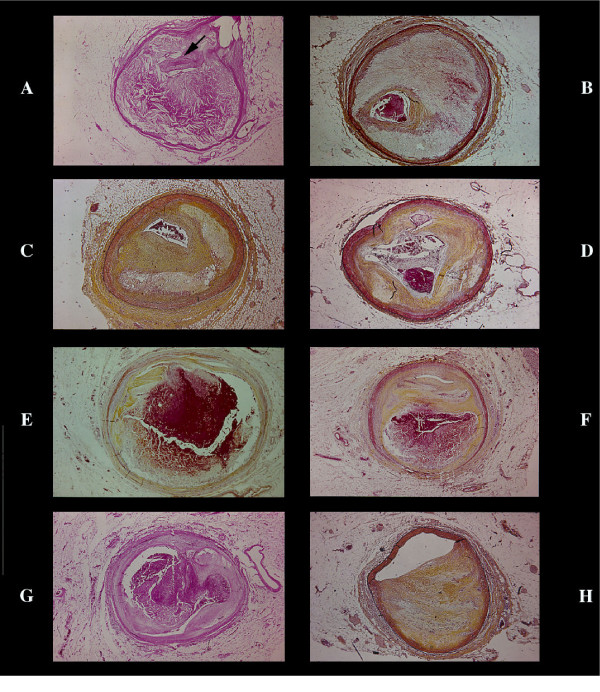
Coronary atherosclerosis. Different aspect of a severe, pin-point lesion (arrow). Plaque with prevailing atheroma (A) or fibrosis (B). Plaque with pale, large zone of proteoglycan accumulation (C) or with small atheroma plus hemorrhage and proteoglycans associated with critical stenosis occluded by an acute thrombus (D). Sequence in the same plaque of "rupture" (E) followed by severe hemorrhagic atheroma with minimal, linear lumen (arrow) without occlusion(F). Occlusive thrombosis connected with hemorrhagic atheromasia at the site of a critical stenosis (G). Semilunar stenosis (H) with a normal half wall and minor lumen reduction. The concept of vessel wall remodeling to compensate plaque growth has not any support (very low frequency of this type of lesion versus severe concentric lumen reduction in the natural history of coronary heart disease).

The recurrent basic changes in myohyperplastic plaque (smooth muscle cell hyperplasia, fibrosis, proteoglycan accumulation with atheroma and/or calcification) explain the various intimal aspects amongst different plaques and different tracts of the same plaque (Fig. 4). A synopsis comparing dogma versus our findings is given in Table 1:

## Targets of ultrasound diagnosis: the present and the future

Different echocardiographic techniques have been employed in the attempt to provide adequate visualization of coronary arteries (Table 2). However, intracoronary ultrasound (ICUS) represents the most valuable method to assess plaque morphology. Given to its limited resolution (approximately 0.3 mm), angiography is a fairly imprecise measure of luminal morphology and size. In particular, it is deficient in providing adequate distinction between plaque and lumen irregularities and assessing the extent of atherosclerotic disease [6]. Both these issues appear to be much well defined by ICUS. Firstly, the tomographic orientation of ultrasound enables a visualization of the full circumference of the vessel wall and, therefore, a more accurate assessment of size [7-9]. In addition, it allow us to overcome the false assumption that the nonstenotic region surrounding a discrete stenosis is normal and, therefore, to obtain an unbiased assessment of the plaque burden at the site of the stenosis. Finally, the penetrating nature of ultrasound provides unique images of the atherosclerotic plaque.

**Table 2:** Echocardiographic approach to coronary arteries.

*Technique*	*% success*	*Image quality*	*Anatomic information (plaque)*	*Functional information (flow)*
Transtoracic	20	±	±	±
Transesophageal	80	+	+	++
Epicardic	90	++	++	-
Intracoronary	95	+++	+++	-

- = poor; + = sufficient; ++ = good; +++ = very good

ICUS image analysis has been extensively used for determining plaque composition [10-13]. However, there are some limitations to this approach: 1) digitizing videotapes is time-consuming and therefore not suitable for real-time analysis; (2) image resolution is reduced to that of videotape, approximately 330 μm; (3) parameters such as gain, including time gain compensation and intensity, can be adjusted by the operator, thereby adding variability to the data set; (4) the dynamic range, pre- and postprocessing of the images depend on the analog-to-digital converters used in the ICUS consoles; (5) finally, due to the small dimension of the transducer, the transmitted acustic energy is low. Thus, some concerns still exist on whether this technique is *ready to go *for clinical use. In particular, definition of plaque composition seems not enough reproducible to provide an alternative independent standard to quantitative histology [14].

New technical development based on noninvasive molecular imaging [15,16], such as the use of novel targeted contrast agents able to identify fibrin deposited within plaque microfissures [17], adhesion or thrombogenic molecules expressed on endothelium of vulnerable plaques [18-22], matrix metalloproteinases in the cores of progressing lesions [23], or even early angiogenic expansion of the vasa vasorum that supports plaque development [24], will contribute to fill the gap between information derived from direct, quantitative histology and ultrasound imaging. Moreover, spectral analysis of the radiofrequency signal allows a more detailed analysis of various vessel components than does image analysis of digitized videotape images and can be potentially employed in real-time. This approach is expected to improve tissue characterization [25].

### Comment

The working hypothesis is to stabilize the atherosclerotic plaque by increasing the thickness of the fibrous cap or by regression of atheroma burden (1). However, present imaging techniques (coronary cineangiography, angioscopy, contrast magnetic resonance, contrast echocardiography, nuclear scintigraphy, etc.) cannot provide adequate clinical evaluation of plaque vulnerability. In particular, ICUS is unable to provide discrimination between physiological and pathological intimal thickening and to define the shape of plaque, i.e. concentric or semilunar. In fact, amongst 2121 coronary sections at the site of maximal lumen-diameter reduction the stenosis was concentric in 70% of the cases (99% in supplying vessels of an acute myocardial infarct). Furthermore, in 408 CHD patients the maximal stenosis in each single case was less than 69% in 68, 70%, in 67, 80% in 109 and >90% in 164. These data mean that the residual lumen ranged from 900 to less than 50 μm and catheter of 1500 μm in most instances must break the plaque, being the residual lumen too small. Therefore, shape and contour of a plaque can be altered with a misleading higher frequency of semilunar stenosis giving an erroneous support to the questionable concept of vessel wall remodelling following an atherosclerotic plaque formation.

According to the previous data the main conclusions are: 1. The natural history of coronary atherosclerotic plaque in the general population, inclusive of CHD patients, is different from plaques obtained by experimental hypercholesterol diet or found in familial hypercholesterolemia. Most data refer to the latter as a valid model for the human plaque. In particular, fatty streaks is not the starting change of the myohyperplastic atherosclerotic plaque; 2. Emphasis is given to a "macrophagic inflammation" as source of proteolytic and/or thrombogenic moleculae causing plaque rupture. However, macrophagic reaction belongs to a repair process to digest necrotic or extraneous material rather than typical elements of an inflammatory process, as lymphocytes, neutrophils. The assumption that on increased number of labelled macrophages may indicate a risk of rupture is questionable in human coronary myohyperplastic plaque.

In the present review we have discussed the behaviour and meaning of components of the human coronary atherosclerotic plaque to emphasize the inconsistency of the current myths:

1. Experimental hypercholesterol model and correspondent human conditions do not represent the natural history of atherosclerosis in coronary arteries in the general population.

2. Physiological intimal thickening can not be interpreted as starting point of the atherosclerotic process.

3. Fatty streak does not represent the early atherosclerotic lesion.

4. Calcification is not synonymous of severe stenosis.

5. Hemorrhage is not consequent to endothelial fissuration.

6. Prevention of macrophage "inflammation" as source of substances able to disrupt the fibrous cap allowing rupture and thrombosis as well as identification of the thickness of fibrous cap to diagnose a vulnerable plaque may have little, if any, sense. Rupture and thrombosis may be secondary phenomena and not the cause of an acute coronary syndrome.

7. Degree and number of severe coronary plaques do not predict onset, course, complications and death in CHD.

## Authors' contributions

Prof. Giorgio Baroldi contributed to the conception and organization of this review and to the final comments.

Dr. Riccardo Bigi and Dr. Lauro Cortigiani summarized the use of ultrasound techniques in atherosclerotic plaque imaging

**Table 1:** Natural History of Human Coronary Atherosclerotic Plaque

**Beliefs**	**Facts**
Transendothelial lipoprotein/cholesterol infiltration	Nodular smooth myocell-elastic hyperplasia protruding in lumen
Fatty streaks	Fibrous substitution
Macrophagic "inflammation"	Proteoglycan accumulation below fibrous cap between media/intima
Necrotic core-atheroma under fibrous cap	Interstitial/macrofagic (foam cells) storage of lipoprotein-cholesterol and/or calcium salts in proteoglycan pool
Rupture fibrous cap	Plaque tridimensional growth by recurrence of previous phenomena
Thrombosis-Embolization	Late vascularization of atherosclerotic intima


Divergency between dogma and heretic view is easily explained by the fact that the former is founded on experimental hypercholesterolemic plaque which more or less may correspond to plaques in the relatively small group of familial hypercholesterolemia in humans. Furthermore, the main arterial vessel examined was the aorta and in human pathology advanced plaque was not studied comparing study in different CHD patterns and pathologic and normal controls.
